# Effect of perioperative ONS combined with intestinal microecology in patients with colorectal cancer: a randomized clinical trial

**DOI:** 10.3389/fnut.2025.1588399

**Published:** 2025-05-27

**Authors:** Xuanjun Liu, Weixu Mao, Guowei Zhao, Juan Liao, Qigang Li, Gan He

**Affiliations:** ^1^Department of Gastrointestinal Surgery, Yongchuan Hospital of Chongqing Medical University, Chongqing, China; ^2^Department of Respiratory Medicine, The Affiliated Yongchuan District Traditional Chinese Medicine Hospital of Chongqing Medical University, Chongqing, China; ^3^Central Laboratory, Yongchuan Hospital of Chongqing Medical University, Chongqing, China

**Keywords:** colorectal cancer, perioperative period, oral nutritional supplements, intestinal microecology, postoperative recovery

## Abstract

**Purpose:**

To investigate the combined effects of oral nutritional supplement (ONS) and intestinal microecology on postoperative nutrition status, inflammatory response and intestinal flora regulation colorectal cancer (CRC) patients.

**Methods:**

This prospective single-center randomized controlled trial (RCT) was conducted at Chongqing Yongchuan Hospital between December 2023 and December 2024. CRC patients were randomly assigned to one of two groups: a control group receiving ONS (55.8 g per dose, three times daily) or a test group receiving ONS (55.8 g per dose, three times daily) combined with bifidobacteria (1.5 g per dose, three times daily).

**Results:**

A total of 62 patients who undergoing radical colorectal cancer resection were enrolled. Participants were equally randomized into control and test groups (*n* = 31 each). At baseline, no significant differences in demographic characteristics, nutritional status, or inflammatory markers were observed between groups (*p* > 0.05). Prealbumin (PA; 174.0 ± 38.0 g/L vs. 149.7 ± 42.9 g/L, *t* = −2.358, *p* = 0.022), albumin (ALB; 36.6 ± 3.3 g/L vs. 33.1 ± 4.0 g/L, *t* = −3.745, *p* < 0.000), total protein (TP; 65.8 ± 5.1 g/L vs. 62.5 ± 6.3 g/L, *t* = −2.266, *p* = 0.027), and the changes in ΔT3–T2 in PA (32.9 ± 36.1 g/L vs. 13.3 ± 34.9 g/L, *t* = −2.180, *p* = 0.033), ALB (4.0 ± 4.5 g/L vs. 1.0 ± 3.7 g/L, *t* = −2.862, *p* = 0.006), and TP (7.5 ± 5.9 g/L vs. 4.0 ± 5.9 g/L, *t* = −2.333, *p* = 0.023) were significantly greater in the test group than in the control group. The reduction in C-reactive protein (CRP) from T2 to T3 (42.1 (27.1, 62.9) mg/L vs. 26.8 (10.7, 46.4) mg/L, Z = −2.752, *p* = 0.006) was significantly greater in the test group. Fecal DNA fingerprint analysis revealed that, compared with the control group, the test group presented significantly greater intestinal flora species richness and abundance. The time to first defecation was significantly shorter in the test group (4.5 ± 1.8 vs. 5.9 ± 1.7 days, *t* = 3.132, *p* = 0.003).

**Conclusion:**

Perioperative ONS combined with intestinal microbiota interventions improves postoperative nutritional status, modulates inflammatory dynamics, and accelerates intestinal function recovery. However, these interventions show limited impact on hospitalization duration and complication rates.

## 1 Introduction

Colorectal cancer (CRC), one of the most common global malignant tumors, exhibits an annually increasing incidence rate and ranks among the leading digestive system causes of death (1). While laparoscopic surgery combined with synchronous chemoradiotherapy is commonly used in CRC clinical treatment, preoperative tumor growth often induces intestinal mucosa ischemia and hypoxia. This disrupts intestinal microecological balance, damages the mucosal barrier, and causes malnutrition (2, 3). Furthermore, preoperative bowel preparation and postoperative fasting exacerbate nutritional deficiencies, leading to reduced immunity, worsening nutritional status, and impaired postoperative recovery. Consequently, CRC treatment strategies have shifted from single-surgery approaches to multidisciplinary, comprehensive perioperative models. The integration of oral nutritional supplements (ONS) and intestinal microecology has emerged as a key research focus.

For patients undergoing radical CRC surgery, malnutrition serves as an independent risk factor for postoperative complications and is closely linked to mortality, prolonged hospitalization, and readmission rates (4). Correcting perioperative malnutrition improves surgical tolerance, reduces postoperative complications, and enhances patient prognosis (5). ONS is the preferred method for delivering supplemental energy and nutrients for special medical purposes beyond regular food (6). Clinical studies and systematic reviews consistently show that ONS improves nutritional status and clinical outcomes in hospitalized patients while also reducing overall costs, underscoring its cost-effectiveness (7, 8). However, ONS alone often fails to address intestinal dysfunction and impaired nutrient absorption in CRC patients, potentially limiting its efficacy.

Intestinal microecosystems have gained significant attention in CRC adjuvant therapy due to their beneficial properties. Studies show that probiotics like *Bifidobacterium* enhance gastrointestinal motility and may slow CRC progression via specific protein and metabolite secretion (9). Recent clinical research indicates that intestinal ecosystem modulation improves perioperative immune indices and postoperative gastrointestinal symptoms in CRC patients while enriching intestinal flora diversity. These interventions effectively correct dysbiosis, accelerate postoperative recovery, and play key roles in CRC prevention and adjuvant treatment (10, 11).

Many existing studies have used single-intervention approaches, limiting CRC clinical management. This study explored the perioperative combination of ONS and intestinal microbial agents. By leveraging intestinal microecology to regulate flora balance and using ONS to supply essential nutrients, the study investigated their combined effects on postoperative recovery, including intestinal flora composition, inflammation, and nutritional status. The goal was to develop more precise and effective CRC treatment strategies.

## 2 Information and methods

### 2.1 Study design

This prospective, single-center, randomized controlled trial enrolled subjects who met the inclusion criteria during the study period. The participants were randomly assigned to either the control or test group via a computer-generated randomization sequence. All the subjects provided written informed consent.

The study was conducted in strict accordance with relevant guidelines and regulations and was approved by the Medical Ethics Committee of Yongchuan Hospital, Chongqing Medical University (Approval No. 2023LLS040).

The inclusion criteria were as follows: (1) patients who underwent elective surgery for CRC and were aged ≥18 years (with no sex restrictions); (2) patients who underwent preoperative colonoscopy biopsy confirming CRC, without multiple primary tumors; (3) patients with no distant metastasis and no preoperative neoadjuvant therapy; (4) patients with no preoperative infections or immunodeficiency diseases and no recent use of nutritional or intestinal microbial preparations; (5) patients with no acute complications, such as intestinal obstruction, intestinal perforation, or gastrointestinal bleeding; and (6) patients who underwent their first laparoscopic radical resection for CRC, with a preoperative nutritional risk screening (NRS2002) score ≥3; (7) patients with normal mental status, with intact acceptance, cognition, judgment, and language communication abilities; and voluntary consent to participate in the study after being informed of its details.

The exclusion criteria were as follows: (1) patients with multiple primary tumors; (2) patients with advanced preoperative or intraoperative tumors or those who received preoperative neoadjuvant therapy; (3) patients with preoperative infections, immunodeficiency diseases, nutritional support therapy, or recent antibiotic use; (4) patients with acute complications, such as intestinal obstruction, perforation, or bleeding; (5) patients with severe immune, cardiovascular, or respiratory diseases; and (6) patients who underwent repeat surgery or who had a preoperative NRS2002 score <3; (7) patients who were allergic to the study drug or were unable to complete the study for other reasons.

### 2.2 Methods

#### 2.2.1 Intervention drugs and ONS

The intestinal microbial agents used in this study were bifidobacterium tetrad live tablets (0.5 g/tablet; Hangzhou Yuanda Biopharmaceutical Co., Ltd.). These agents are composed of the following strains: *Bifidobacterium infantis*, *Lactobacillus acidophilus*, *Enterococcus faecalis*, and *Bacillus cereus*.

The ONS product used was *ENSURE TP* (400 g/tub; Abbott Laboratories B.V.), a balanced nutritional supplement providing approximately 450 kcal of energy, 15.9 g of protein, 15.9 g of fat, 60.7 g of carbohydrates, vitamins, and minerals per 100 g.

### 2.3 Patient management

Both groups were fed a liquid diet beginning on the day of enrollment. The control group received oral enteral nutrition powder (55.8 g per dose, three times per day) until 1 day before surgery (average duration: 4 days). The test group received the same regimen as the control group, with an additional 1.5 g of Bifidobacterium per dose, three times per day.

On the night before surgery, all enrolled patients received polyethylene glycol electrolyte powder (3,000 mL). Prophylactic antibiotics were administered 30 min before surgery, with additional doses given over the next three hours. Surgical procedures followed the China Code for Diagnosis and Treatment of Colorectal Cancer (2023 edition) (12) and were performed by the same surgical team.

In the postoperative period, both groups received routine intravenous nutritional support on days 1 and 2. Patients were allowed to drink small amounts of water on day 2, provided that there was no discomfort (nausea, vomiting, abdominal distension, or abdominal pain). On day 3, patients began taking a small amount of oral enteral nutrition powder (55.8 g per day). On day 4, the dose was increased to 55.8 g twice daily, and on day 5, the regimen was adjusted to 55.8 g three times daily until discharge. For the control group, bifidobacterium (1.5 g per dose, three times daily) was added starting on day 3.

The daily energy requirements for all patients were 25 to 30 kcal/kg/day, and the protein requirements were 1.0 to 2.0 g/kg/day. ONS and parenteral nutrition (PN) were initiated on postoperative day 3. The PN energy supply gradually decreased as the ONS energy supply increased. Intravenous fluid intake was maintained at 2000 to 2,500 mL for the first 2 days and then gradually decreased on the basis of the patient’s ONS intake from day 3 onward.

### 2.4 Study endpoints

#### 2.4.1 Primary study endpoints

The primary endpoints of the study were postoperative nutritional measures, including prealbumin (PA), albumin (ALB), total protein (TP), and hemoglobin (HGB). These parameters were measured on the enrollment day (T1), postoperative day 1 (T2), and postoperative day 8 (T3).

#### 2.4.2 Secondary study endpoints

The secondary endpoints of the study were postoperative inflammatory markers and fecal bacterial DNA fingerprints.

##### 2.4.2.1 Inflammatory indicators

Blood samples were collected at T1, T2, and T3. The inflammatory markers measured included white blood cell count (WBC), lymphocyte count (Lym), neutrophil count (Neu), and C-reactive protein (CRP).

##### 2.4.2.2 Fecal bacterial DNA fingerprint analysis

In this study, 10 fecal samples (3 g each) were randomly selected from the first postoperative stools of both groups and stored at −80°C. For analysis, the fecal samples were processed as follows:

For sample preparation, 100–200 mg of each stool sample was weighed, Buffer SLA, Proteinase K, and grinding beads were added. The mixture was incubated at 70°C for 10 min, followed by centrifugation at 13,000 rpm for 1 min. **DNA Extraction:** RNase A, Buffer SLB, and Buffer SGB were added sequentially. Multiple centrifugation and washing steps were performed. Finally, the genomic DNA was eluted with TE buffer. PCR amplification: The primers PS2 (5′-TG(C/T)ACACACCGCCCGT-3′) and PL2 (5′-GGGT(G/C/T)CCCCCATTC(A/G)G-3′) were used for specific amplification. The PCR protocol included predenaturation at 98°C for 2 min; 30 cycles of denaturation at 98°C for 20 s, annealing at 55°C for 20 s, and extension at 72°C for 10 s; a final extension at 72°C for 5 min; and cooling at 16°C for 2 min. **Product analysis:** The amplified products were analyzed via electrophoresis on a 2% agarose gel, visualized under UV light, and photographed to preserve the results.

#### 2.4.3 Clinical observation indicators

The number and incidence of operation time and postoperative complications (such as lung infection, urinary tract infection, and abdominal infection), postoperative bowel function recovery (measured by time to first postoperative defecation), blood albumin injection utilization, postoperative hospitalization duration, and associated hospitalization costs were recorded.

### 2.5 Sample size calculation

The primary outcome measures in this study were postoperative nutritional indicators, specifically prealbumin levels. In the current literature, the mean prealbumin levels in the test group and control group were reported to be 288.4 ± 58.6 and 239.8 ± 71.8, respectively (13). With a significance level (*α*) of 0.05 and a power (1−*β*) of 0.8, the required sample size for each group was calculated to be 30 cases via PASS software. Considering the short follow-up period and low risk of loss to follow-up in this study, a total of 60 cases (30 per group) were deemed sufficient. This study is a single-center, randomized controlled trial with a minimum of 30 patients in each group (test and control).

### 2.6 Statistical analysis

Statistical analyses were performed via SPSS 26.0 software. A two-sided *p* value of less than 0.05 was considered statistically significant. Normally distributed continuous data are presented as the means ± standard deviations (Means ± SD), whereas nonnormally distributed data are reported as medians and interquartile ranges (M [P25, P75]). Repeated measures analysis of variance (ANOVA), independent samples *t* tests, or Mann–Whitney U tests were used as appropriate. Categorical data are expressed as counts and proportions [*n*, (%)] and were analyzed via the χ^2^ test or Fisher’s exact test, depending on the sample size.

## 3 Results

### 3.1 Comparison of the baseline data between the two patient groups

**Figure 1:**
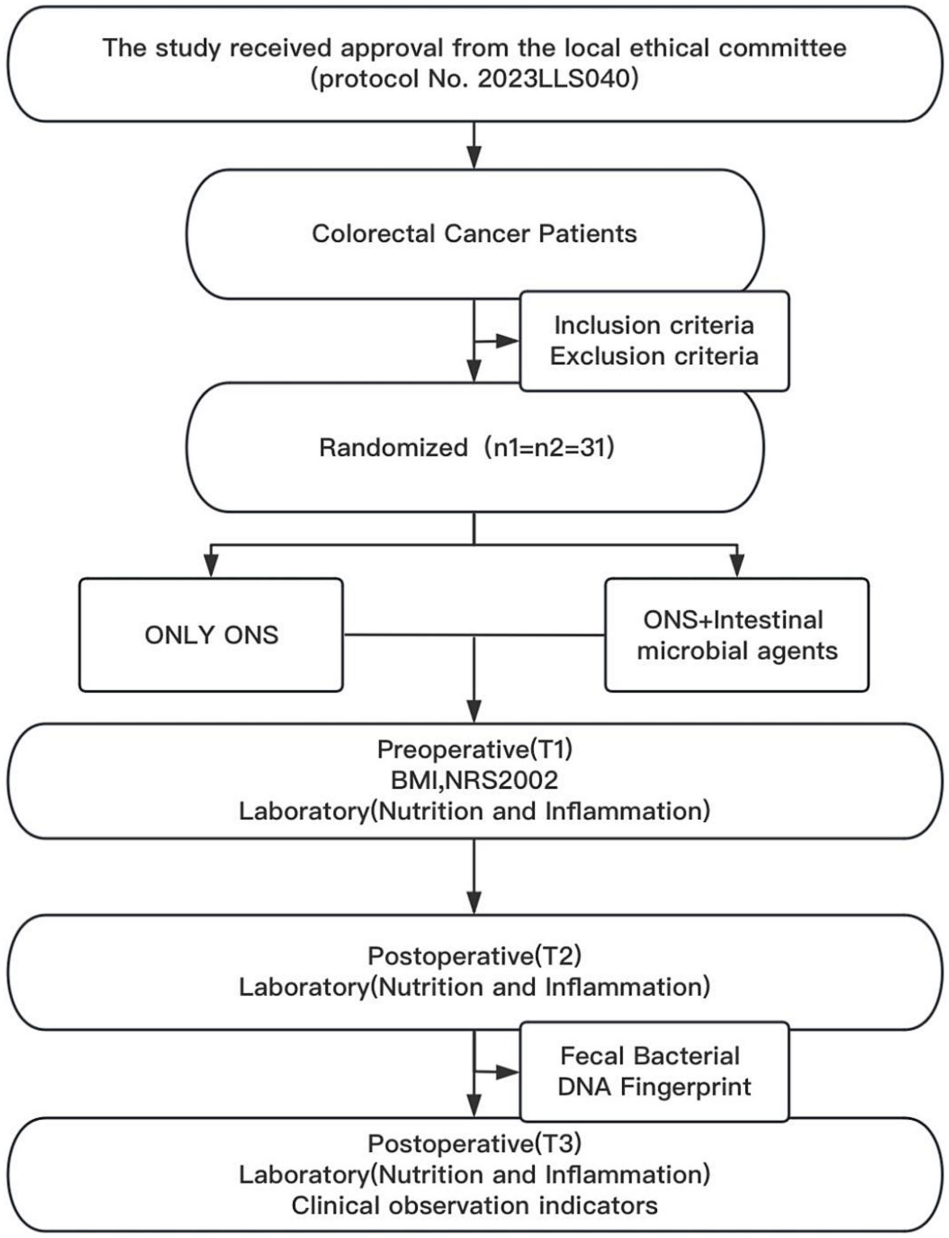
Flow chart of this randomized clinical trial.

A total of 62 patients who underwent radical CRC surgery were included in the study and randomized into control and trial groups (*n* = 31 per group) (Figure 1). Demographic analysis revealed no significant differences between the groups in terms of sex composition (male: 14 vs. 17; female: 17 vs. 14), age (66.8 ± 13.0 years vs. 64.7 ± 10.3 years; t = 0.703, *p* = 0.485), or BMI (22.98 ± 3.15 kg/m^2^ vs. 23.06 ± 2.91 kg/m^2^; t = −0.106, *p* = 0.916).

In terms of oncological characteristics, tumor anatomical location distribution (right colon: 9 vs. 7; left colon: 4 vs. 3; sigmoid colon: 3 vs. 5; rectum: 15 vs. 16 cases; χ^2^ = 0.925, *p* = 0.859), maximum tumor diameter (4.7 ± 1.7 cm vs. 4.0 ± 1.7 cm; t = 1.571, *p* = 0.121), and AJCC 8th Edition TNM stage (stage I: 5 vs. 8 cases; stage II: 14 vs. 11 cases; stage III: 12 vs. 12 cases; χ^2^ = 1.052, *p* = 0.647) were all balanced between groups (all *p* > 0.05). These results confirmed the validity of the randomization and the matching of the baseline characteristics (Table 1).

**Table 1:** Patient baseline data and tumor characteristics.

Variables	Control (*n* = 31)	Test (*n* = 31)	t/χ^2^	*p*-value
Age (in years)	66.8 ± 13.0	64.7 ± 10.3	0.703	0.485
Sex [*n* (%)]				
Male	14 (45.2%)	14 (45.2%)	/	/
Female	17 (54.8%)	17 (54.8%)		
BMI	22.98 ± 3.15	23.06 ± 2.91	−0.106	0.916
NRS2002	3.26 ± 0.44	3.29 ± 0.46	−0.280	0.780
Underlying diseases [*n* (%)]				
Hypertension	9 (29.0%)	5 (16.1%)	1.476	0.363
Diabetes	3 (9.7%)	3 (9.7%)	/	/
Coronary heart disease	1 (3.2%)	1 (3.2%)	/	/
Location				
Right colon	9	7	0.925	0.859
Left colon	4	3		
Sigmoid colon	3	5		
Rectum	15	16		
Maximum diameter	4.7 ± 1.7	4.0 ± 1.7	1.571	0.121
AJCC				
I	5	8	χ^2^ = 1.052	0.647
II	14	11		
III	12	12		

### 3.2 Comparison of nutritional and inflammatory indicators between the two groups

A repeated-measures two-factor analysis of variance (group × time) was conducted. The time main effect revealed that all nutritional indicators (PA, ALB, TP, HGB) and inflammatory indicators (CRP, WBC, etc.) exhibited statistically significant changes over time (*p* < 0.05). These findings suggest substantial dynamic fluctuations in nutritional and inflammatory status during postoperative recovery. The interaction effect showed that the time × group interaction for CRP was significant (*F* = 3.298, *p* = 0.04), indicating that the intervention differentially regulated the dynamic trajectory of CRP. No significant interaction effects were detected for the other indicators (*p* > 0.05). The main effect between groups was not significant for CRP (*F* = 0.363, *p* = 0.549) or other indicators (*p* > 0.05), indicating that the groups were comparable at baseline (Table 2).

**Table 2:** Nutritional and inflammatory indicators of the two groups.

Variables	Group	T1	T2	T3	Intergroup (F, P)	Time (F, P)	Interaction (F, P)
PA (g/L)	Control (*n* = 31)	204.5 ± 43.2	136.5 ± 36.8	149.7 ± 42.9	2.435	135.043	2.369
	Test (*n* = 31)	218.5 ± 49.7	141.1 ± 37.1	174.0 ± 38.0^*^	0.124	0.000	0.098
ALB (g/L)	Control (*n* = 31)	39.0 ± 3.9	32.2 ± 3.7	33.1 ± 4.0	3.775	62.212	3.248
	Test (*n* = 31)	39.9 ± 6.7	32.7 ± 3.8	36.6 ± 3.3^*^	0.057	0.000	0.077
TP (g/L)	Control (*n* = 31)	69.4 ± 6.3	58.5 ± 6.0	62.5 ± 6.3	1.301	116.491	2.786
	Test (*n* = 31)	70.4 ± 5.7	58.3 ± 5.2	65.8 ± 5.1^*^	0.259	0.000	0.066
HGB (g/L)	Control (*n* = 31)	122.2 ± 22.3	116.9 ± 18.2	117.2 ± 22.7	1.468	6.935	1.632
	Test (*n* = 31)	127.1 ± 20.1	117.9 ± 17.4	123.8 ± 20.2	0.230	0.011	0.206
CRP (mg/L)	Control (*n* = 31)	3.3 (1.3, 11.3)	68.5 (39.0, 97.8)	32.5 (11.4, 63.8)	0.363	101.192	3.298
	Test (*n* = 31)	3.0 (1.4, 8.2)	74.1 (48.1, 89.9)	21.6 (14.4, 31.8)	0.549	0.000	0.040
WBC (10^9^/L)	Control (*n* = 31)	5.8 ± 1.6	10.4 ± 2.6	6.8 ± 2.6	0.047	103.902	0.773
	Test (*n* = 31)	6.1 ± 1.9	9.9 ± 3.0	6.7 ± 2.1	0.830	0.000	0.464
Lym (10^9^/L)	Control (*n* = 31)	1.4 ± 0.5	1.0 ± 0.4	1.2 ± 0.4	0.012	25.529	0.099
	Test (*n* = 31)	1.5 ± 0.9	0.9 ± 0.4	1.2 ± 0.4	0.913	0.000	0.754
Neu (10^9^/L)	Control (*n* = 31)	3.9 ± 1.3	8.8 ± 2.5	4.9 ± 2.4	0.037	135.924	0.978
	Test (*n* = 31)	4.2 ± 1.3	8.3 ± 2.7	4.9 ± 1.7	0.848	0.000	0.379

*p < 0.05, test group vs. control group.

**Figure 2:**
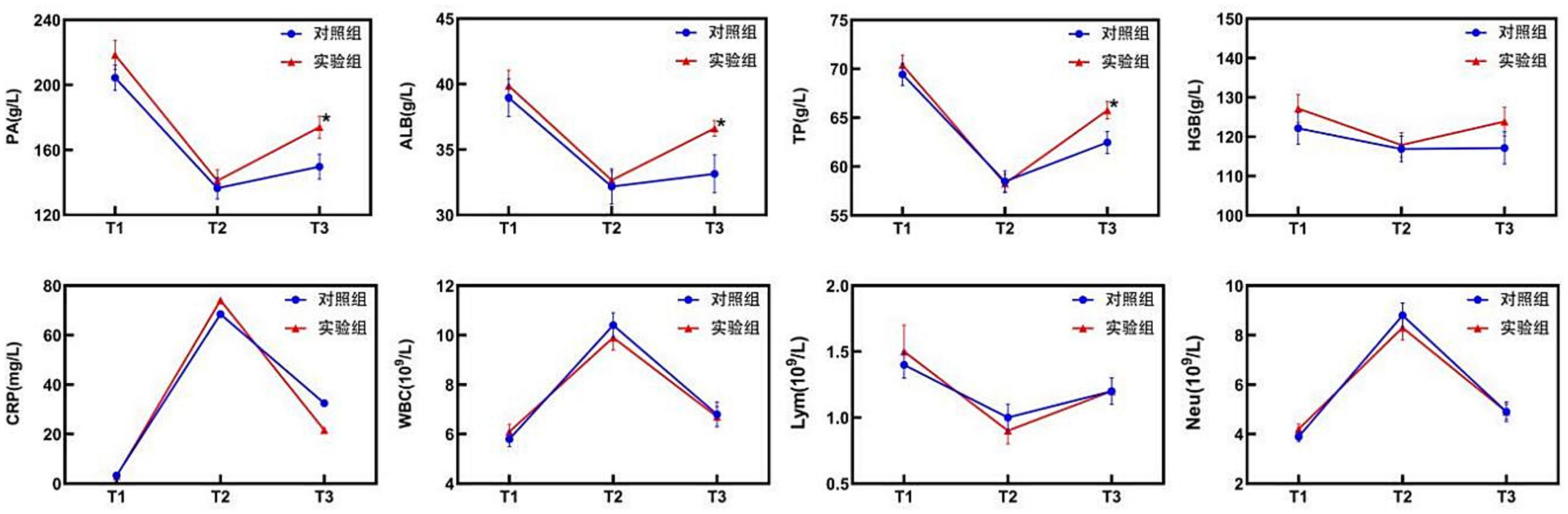
Changes in nutritional and inflammatory indicators in the two groups. CRP levels are presented as the median (upper/lower limit); ^*^ T3, test group vs. control group, *p* < 0.05.

*Post hoc* analyses of nutritional and inflammatory indicators at different time points and change values revealed no significant differences between the two groups (*p* > 0.05), confirming good baseline balance and comparability. At T2, the levels of nutritional indicators (PA, ALB, TP, HGB) and Lym significantly decreased compared with those at T1, whereas the levels of inflammatory markers (CRP, WBC) increased with stress. However, these differences were not statistically significant between the groups (*p* > 0.05). At T3, the test group exhibited superior nutritional recovery compared with the control group, with significant increases in PA (174.0 ± 38.0 vs. 149.7 ± 42.9 g/L; *p* = 0.022), ALB (36.6 ± 3.3 vs. 33.1 ± 4.0 g/L; *p* < 0.001), and TP (65.8 ± 5.1 g/L vs. 62.5 ± 6.3 g/L; *p* = 0.027) (Table 2; Figure 2). Analysis of the change values revealed that, compared with the control group, the test group presented a significantly greater rebound in the ALB concentration (4.0 ± 4.5 vs. 1.0 ± 3.7 g/L; *p* = 0.006). Additionally, the rate of CRP decline was faster in the test group (42.1 vs. 26.8 mg/L; *p* = 0.006), indicating that the combined intervention accelerated postoperative inflammation resolution and nutritional recovery (Table 3). ΔT2–T1 and ΔT3–T1: No significant differences were observed between groups during the surgical stress period (T1–T2) or over the full recovery period (T1–T3) (*p* > 0.05).

**Table 3:** Changes in nutritional and inflammation indicators between the two groups.

Variables	Change	Control (*n* = 31)	Test (*n* = 31)	t/Z	*p*-value
RA (g/L)	ΔT2–T1	68.0 ± 34.4	77.5 ± 36.6	−1.051	0.239
	ΔT3–T1	54.7 ± 31.8	44.5 ± 39.3	1.123	0.627
	ΔT3–T2	13.3 ± 34.9	32.9 ± 36.1	−2.180	0.033^*^
ALB (g/L)	ΔT2–T1	6.8 ± 4.0	7.2 ± 6.3	−0.322	0.517
	ΔT3–T1	5.8 ± 3.8	3.2 ± 6.8	1.834	0.617
	ΔT3–T2	1.0 ± 3.7	4.0 ± 4.5	−2.862	0.006^*^
TP (g/L)	ΔT2–T1	11 ± 6.7	12.1 ± 5.7	−0.744	0.532
	ΔT3–T1	7 ± 5.9	4.6 ± 5.5	1.612	0.877
	ΔT3–T2	4.0 ± 5.9	7.5 ± 5.9	−2.333	0.023^*^
HGB (g/L)	ΔT2–T1	5.3 ± 12	9.2 ± 12	−1.303	0.165
	ΔT3–T1	5 ± 13.1	3.3 ± 13	0.515	0.826
	ΔT3–T2	0.3 ± 9.2	5.9 ± 9.7	−2.361	0.021^*^
CRP (mg/L)	ΔT2–T1	58.5 (33.5, 85.0)	63.9 (41.5, 83.5)	−1.049	0.294
	ΔT3–T1	28.7 (10.1, 61.5)	14.4 (7.6, 30.2)	−1.260	0.208
	ΔT3–T2	26.8 (10.7, 46.4)	42.1 (27.1, 62.9)	−2.752	0.006^*^
WBC (10^9^/L)	ΔT2–T1	4.5 ± 2.5	3.8 ± 2.7	1.139	0.582
	ΔT3–T1	0.9 ± 2.5	0.6 ± 2	0.523	0.482
	ΔT3–T2	3.6 ± 2.7	3.1 ± 1.9	0.765	0.447
Lym (10^9^/L)	ΔT2–T1	0.5 ± 0.4	0.5 ± 0.9	−0.223	0.913
	ΔT3–T1	0.2 ± 0.3	0.3 ± 0.7	−0.428	0.873
	ΔT3–T2	0.3 ± 0.4	0.2 ± 0.4	0.252	0.802
Neu (10^9^/L)	ΔT2–T1	5.0 ± 2.5	4.2 ± 2.6	1.260	0.213
	ΔT3–T1	1.0 ± 2.4	0.7 ± 1.7	0.563	0.576
	ΔT3–T2	4.0 ± 2.7	3.5 ± 1.9	0.877	0.384

*p < 0.05, test group vs. control group.

### 3.3 Comparison of fecal bacterial DNA fingerprint analysis between the two groups

**Figure 3:**
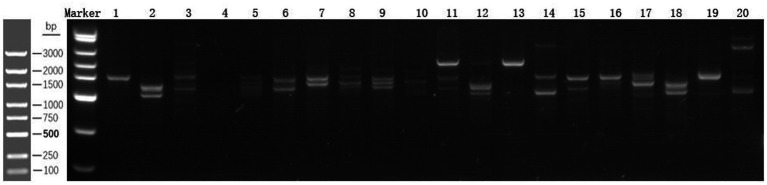
Fecal bacterial DNA fingerprint analysis, control groups 1–10, and test groups 11–20.

According to the map analysis, the test group presented an increase in band intensity compared with the control group, indicating a greater number of intestinal bacteria. This suggests an increase in both species richness and abundance, as well as a significant change in the diversity of the intestinal flora (Figure 3).

### 3.4 Clinical observation indicators in both groups

Clinical observation indicators included operation time, albumin supplementation, hospital fees, postoperative hospital stay, time to first postoperative defecation, and incidence of postoperative complications. The defecation time was significantly shorter in the test group than in the control group (*p* < 0.05). However, no significant differences were observed in other indicators, including human ALB injection use, hospitalization cost, and incidence of postoperative complications (Table 4).

**Table 4:** Clinical observations of the two groups.

Variables	Control (*n* = 31)	Test (*n* = 31)	t/Z/χ^2^	*p*-value
Operation time (h)	249.7 ± 71.6	224.9 ± 62.4	1.452	0.152
Albumin supplementation (vial)	0 (0, 6)	0 (0, 4)	−1.330	0.184
Hospital fees (thousand RMB)	53.4 ± 10.4	51.1 ± 9.3	0.932	0.700
Postoperative hospital stay (d)	11.9 ± 5.6	10.8 ± 1.5	1.076	0.290
Defecation time	5.9 ± 1.7	4.5 ± 1.8	3.132	0.003^*^
Total postoperative complications [*n*, (%)]	2 (6.6%)	1 (3.3%)	0.357	0.550

*p < 0.05, test group vs. control group.

## 4 Discussion

The development of CRC is associated with imbalances in nutritional metabolism and the intestinal flora. This association presents dual challenges for perioperative management: addressing insufficient nutrient supply and mitigating flora-mediated immunosuppression. Compared with normal cells, tumor cells exploit the host’s glucose metabolism via the Warburg effect, increasing their glycolysis rate 20- to 30-fold. This results in a high catabolic state (14). However, surgical trauma exacerbates metabolic disorders, and intestinal ischemia–reperfusion injury increases intestinal epithelial cell apoptosis. This triggers intestinal barrier dysfunction and enhances the translocation of gram-negative bacteria, leading to a vicious cycle of endotoxemia and systemic inflammation (3, 15, 16). In this context, traditional single-intervention models, such as simple enteral nutrition or antibiotics, often fail to address these interconnected issues comprehensively.

ONS are specialized nutritional formulations designed to provide patients with energy, protein, and micronutrients. They are widely used in pre- and postoperative care, for patients with chronic wasting diseases, and among elderly populations at nutritional risk. The primary advantages of ONS include rapid energy replenishment, the ability to mitigate the negative nitrogen balance, and reduced muscle catabolism. These effects help maintain organ function and immune status, thereby improving the overall nutritional health of patients (17). In 2020, the European Society for Parenteral and Enteral Nutrition (ESPEN) recommended preoperative ONS use regardless of the patient’s nutritional status, as the clinical benefits outweigh potential surgical risks (18). However, ONS focuses primarily on the macronutrient supply and lacks targeted regulation of the intestinal microecology. This limitation makes it difficult to interrupt the inflammatory cascade triggered by intestinal dysbiosis, which can persist postoperatively or during disease states.

In recent years, increasing evidence has highlighted a causal link between gut microbial dysbiosis and cancer development. Specific pathogens, such as *Fusobacterium nucleatum*, *anaerobic Streptococcus*, and enterotoxin-producing *Bacteroides fragilis*, have been shown to promote CRC development through mechanisms including tumor proliferation, inflammation, DNA damage, and immune evasion (19, 20). Consequently, modulating the gut microbiota to restore microbial homeostasis has emerged as a promising strategy for CRC prevention and treatment. Gut microbial agents are biological preparations that enhance host health by regulating the composition or function of the intestinal flora. These agents primarily include probiotics, prebiotics, and synbiotics. The mechanisms of different probiotic strains vary on the basis of their specific activities. Its clinical value lies in restoring the intestinal microecological balance by restoring dominant bacterial populations and inhibiting inflammatory pathways (21). However, colonization efficiency is significantly reduced when the host lacks sufficient metabolic substrates.

In the present study, we innovatively applied a combination of bifidobacterium tetrad live bacterial tablets (*Bifidobacterium infantis*, *Lactobacillus acidophilus*, *Enterococcus faecalis*, and *Bacillus cereus*) and *ENSURE TP* (containing 15.9 g protein per 100 g) in the perioperative management of CRC. The results revealed that the serum ALB levels on postoperative day 8 (36.6 ± 3.3 vs. 33.1 ± 4.0 g/L; *p* < 0.001), total protein levels (65.8 ± 5.1 vs. 62.5 ± 6.3 g/L; *p* = 0.027), and prealbumin levels (174.0 ± 38.0 vs. 149.7 ± 42.9 g/L; *p* = 0.022) were significantly greater in the test group than in the control group. These findings suggest that the combined intervention overcomes the limitations of traditional single interventions, thereby improving patients’ nutritional status and promoting postoperative recovery.

Moreover, fecal DNA fingerprint analysis of the ITS region revealed that, compared with the control group, the test group presented increased bacterial richness and abundance. Specifically, the numbers of intestinal *Bifidobacterium* (*p* = 0.017) and *Enterococcus* (*p* = 0.02) increased, as did the abundances of the four commensal microbiota genera previously reported by Xie et al. (10). This effect may be attributed to the dual mechanism of short-chain fatty acids (SCFAs) provided by *ENSURE TP* (22, 23): (1) SCFAs serve as the primary energy substrate for colonic epithelial cells, enhancing amino acid and micronutrient transport efficiency, and (2) *Bifidobacterium* upregulates the expression of the tight junction proteins ZO-1 and Occludin, thereby strengthening the structural integrity of the tight junction complex between intestinal epithelial cells and reducing intestinal mucosal permeability. Collectively, these mechanisms establish a positive cycle of “microbiota metabolism–intestinal barrier repair–nutrient absorption”. Although Feijo et al. (24) reported no improvement in ALB levels with *ω*-3 fatty acid-enriched ONS in gastric cancer patients undergoing chemotherapy, and a meta-analysis by Rinninella et al. (25) found no significant differences in the serum ALB concentration (*p* > 0.05). Our study demonstrated that the dual-axis strategy of nutrient supply–microbiota metabolic regulation reversed the deterioration of nutritional indicators and confirmed the synergistic benefits of the combined intervention. Future research should employ metagenomic sequencing and metabolomics to further elucidate the interaction mechanisms between the functional microbiota and host metabolism.

Perioperative stress induces proinflammatory stimuli in patients, increasing intestinal mucosal permeability. The intestinal microecology can mitigate intestinal inflammation and repair the intestinal epithelial barrier by reducing cytokine release (26). In this study, although the temporal trends of WBC, Neu, and CRP initially decreased (with Lym decreasing before increasing), the differences in WBC, Neu, Lym, and CRP were not significant (*p* > 0.05). This aligns with Feijo et al.’s (24) neutral conclusion on the effect of *ω*-3-enriched ONS on the CRP but contradicts Niu et al. (27). A meta-analysis revealed that perioperative immunonutrition significantly reduced WBC and CRP levels in gastrointestinal cancer patients.

Notably, in the analysis of dynamic changes in inflammatory markers, the decrease in CRP from T2 to T3 was 42.1 mg/L in the test group, which was significantly greater than the 26.8 mg/L observed in the control group (*p* = 0.006). Similarly, a randomized controlled study by Park et al. (28), which included 73 CRC patients who received oral prebiotics 7 days before surgery, revealed that the inflammatory markers IL-6 and CRP were significantly higher in the test group than in the control group. Mechanistic studies suggest that live bifidobacterium tetrad tablets may coregulate the inflammatory response through multiple pathways. *Lactobacillus acidophilus* can competitively inhibit pathogenic bacteria (e.g., *Escherichia coli*), reducing endotoxin release and inhibiting the activation of the MAPK and NF-κB signaling pathways (29). Indole-3-lactate, produced by *Bifidobacterium infantis* via tryptophan metabolism, activates AhR receptors, thereby inhibiting NF-κB signaling pathway activity (30, 31). A moderate amount of linoleic acid in *ENSURE TP* can accelerate the inflammatory phase of wound healing, reduce the inflammatory response, and promote wound proliferation and remodeling (32). Thus, the combined application of ONS and microbial agents can create a dual-coordination strategy of “nutrient supply–microflora regulation”, overcoming the metabolic and microecological limitations of single interventions.

Despite positive results in terms of nutritional and inflammatory measures, no significant differences were observed between the two groups in terms of hospital length of stay (10.8 ± 1.5 vs. 11.9 ± 1.9 days) or complication rates (12.9 vs. 16.1%). Similar to the findings of Lee SY et al. (33), patients receiving oral arginine-rich ONS and *ω*-3 fatty acids for 7 days showed no significant differences in morbidity (31.6 vs. 29.3%; *p* = 0.743) or length of stay (7.6 ± 2.5 vs. 7.4 ± 2.3 days; *p* = 0.635). However, a meta-analysis of multiple randomized controlled trials (RCTs) confirmed the value of prebiotics and probiotics in reducing postoperative complications, particularly infectious complications (34). This discrepancy may be related to insufficient statistical power due to the small sample size in our study. Additionally, we observed accelerated recovery of intestinal function in the test group, with a shorter time to first postoperative defecation than in the control group (4.5 ± 1.8 vs. 5.9 ± 1.7 days; *p* = 0.003). Similar findings were reported by Wang et al. (35), who demonstrated that early enteral nutrition can shorten the time to first defecation in CRC patients (*p* < 0.001) and promote postoperative intestinal recovery.

In conclusion, our study demonstrated that perioperative ONS combined with intestinal microecological interventions in CRC patients significantly improved nutritional status (e.g., increased levels of PA, ALB, and TP), regulated the inflammatory process (as evidenced by reduced CRP levels), and accelerated gastrointestinal recovery (shorter time to first postoperative defecation). However, several limitations should be acknowledged. First, the single-center study design may introduce selection bias, and the small sample size limits statistical power. Second, the mechanistic insights are insufficient, with a lack of key functional biomarkers such as SCFAs, ZO-1, and Occludin. Third, the short observation period (30 days) precludes a comprehensive evaluation of long-term outcomes, including 90-day postoperative complications and long-term prognosis indicators (e.g., 3-year disease-free survival rate, overall survival rate, and tumor recurrence and metastasis rates). Future research should focus on the following directions: utilizing metagenomics technology to analyze the functional genomic characteristics of key microbiota, such as *Bifidobacterium infantis* and *Lactobacillus acidophilus*, precisely. Multicenter randomized controlled trials (RCTs) should be conducted to comprehensively evaluate the cost-effectiveness of the intervention program and its impact on optimizing medical resource utilization.

## 5 Conclusion

Perioperative ONS combined with intestinal microecological interventions can effectively increase serum PA, ALB, and TP levels in patients who have undergone CRC surgery. This combination therapy has synergistic effects on improving postoperative nutritional status, alleviating inflammatory responses, and promoting the recovery of gastrointestinal function.

## Data Availability

The raw data supporting the conclusions of this article will be made available by the authors, without undue reservation.
